# When controls are healthy: the difficulty of testing neuroprotection in low-injury models

**DOI:** 10.1186/s40635-026-00868-7

**Published:** 2026-02-25

**Authors:** Wilhelm Behringer, Benjamin Abella, Jasmin Arrich, Bernd Boettiger, Michael Holzer

**Affiliations:** 1 Department of Emergency Medicine, Medical University of Vienna, Vienna, Austria; 2 Department of Emergency Medicine, Icahn School of Medicine at Mount Sinai, New York, USA; 3 Medical Faculty, University of Cologne, Cologne, Germany; 4 Institute of Emergency Care, Pedagogy of Health and Nursing Sciences, University of Pécs, Pecs, Hungary

Dear Editor,

We read with great interest the article by Persson et al. examining the effects of hypothermia timing after cardiac arrest in a porcine model [1]. While we commend the authors for their rigorous experimental design, comprehensive outcome assessments, and attention to animal welfare, we have significant concerns regarding the interpretation and presentation of the study's findings.

The authors conclude that "This study did not demonstrate a beneficial effect of hypothermia, whether initiated immediately or two hours after ROSC, compared with normothermia on histological or functional outcomes in adult-sized swine." However, this conclusion is potentially misleading given a fundamental limitation: the control group exhibited near-normal histological and functional outcomes.

When baseline injury severity in the control group is minimal, therapeutic interventions have limited opportunity to demonstrate beneficial effects. This represents a classic "floor effect" where outcome measures are already at or near optimal values, leaving no room for improvement. The absence of demonstrable treatment effect under these conditions does not constitute evidence against hypothermia efficacy; rather, it indicates the experimental model failed to produce sufficient brain injury to adequately test the hypothesis.

We respectfully suggest the authors' conclusion should be revised to more accurately reflect the study's actual findings: "The brain insult in our swine model was not severe enough to test our hypothesis regarding the neuroprotective effects of hypothermia. The near-normal histological and functional outcomes observed in the control group precluded the ability to detect potential beneficial effects of hypothermia initiated immediately or two hours after ROSC."

This distinction is crucial for these several important reasons:

- Clinical translation: The current conclusion may be misinterpreted as evidence against therapeutic hypothermia in cardiac arrest, potentially inappropriately influencing future clinical practice guidelines and treatment protocols.
- Scientific accuracy: The conclusion should clearly distinguish between "no effect demonstrated due to inadequate injury model" versus "no therapeutic effect exists"—these represent fundamentally different scientific interpretations with distinct implications.
- Research direction: Acknowledging insufficient injury severity highlights the critical need for model optimization rather than dismissing hypothermia's therapeutic potential, thus appropriately guiding future experimental design.
- Contemporary context: Current guidelines on temperature management remain conflicting [2–5]; therefore, it is essential to accurately characterize preclinical findings to avoid compounding existing confusion in the field.

Importantly, the study's biomarker findings support this interpretation. Neurofilament light-chain (NfL) levels were significantly lower in the immediate hypothermia group at 48 h and day 7 compared to normothermia. This suggests that even in a model with limited injury, neuroprotective effects may exist that warrant further investigation using a more robust injury paradigm.

We believe revising the conclusions to accurately reflect the limitation of insufficient injury severity would significantly enhance the scientific integrity of this important work and provide more appropriate and valuable guidance for future research efforts, while preserving the study's valuable methodological contributions.

## Data Availability

Not applicable.
